# Social Vulnerability Index and Cardiovascular Disease Care Continuum: A Scoping Review

**DOI:** 10.1016/j.jacadv.2024.100858

**Published:** 2024-03-06

**Authors:** Ramzi Ibrahim, Enkhtsogt Sainbayar, Hoang Nhat Pham, Mahek Shahid, Ahlam A. Saleh, Zulqarnain Javed, Safi U. Khan, Sadeer Al-Kindi, Khurram Nasir

**Affiliations:** a Department of Medicine, University of Arizona-Tucson, Tucson, Arizona; b Health Sciences Library, University of Arizona-Tucson, Tucson, Arizona; c Division of Cardiovascular Prevention and Wellness, Department of Cardiology, Houston Methodist Debakey Heart and Vascular Center, Houston, Texas, USA; d Center for Cardiovascular Computational and Precision Health, Houston Methodist, Houston, Texas, USA; e Houston Methodist Academic Institute, Houston Methodist, Houston, Texas, USA

**Keywords:** cardiovascular disease, disparities, social determinants, social vulnerability index

## Abstract

**Background:**

Social vulnerability index (SVI) estimates the vulnerability of communities to disasters, encompassing 4 separate domains (socioeconomic, household composition and disability, minority status and language, and housing and transportation). The SVI has been linked with risk and outcomes of cardiovascular disease (CVD).

**Objectives:**

This scoping review explored the literature between the SVI and CVD continuum, with a goal to identify gaps in understanding the impact of the SVI on CVD and to elucidate future research opportunities.

**Methods:**

We systematically searched 7 databases from inception to May 19, 2023, for articles that explored the relationship between the SVI and CVD care continuum, including prevention, diagnosis and prevalence, treatment, and health outcomes. Extracted data included SVI ranking type, populations, outcomes, and quality of studies.

**Results:**

Twelve studies evaluated the impact of SVI on the CVD continuum. Five studies explored mortality outcomes, 3 studies explored CVD risk factor prevalence, 4 studies explored CVD prevalence, 1 study explored access to health care in those with CVD, 1 study explored the use of cardiac rehabilitation services, and 1 study explored heart failure readmission rates, all of which revealed statistically significant associations with SVI. All studies included the SVI aggregate percentile ranking, while 5 studies focused on individual thematic components. We identified gaps in understanding the SVI's impact on CVD care continuum, particularly regarding CVD prevention and early detection.

**Conclusions:**

This review provides a comprehensive understanding of the SVI's application in assessing various aspects of the CVD care continuum and highlights potential avenues for future research.

Cardiovascular disease (CVD) continues to pose a major health challenge across the globe, with the United States being no exception. Nearly 50% of American adults experience CVD to varying extents^1^ and CVD claims more lives than all types of cancer combined in the United States.^1^ The burden of this disease is heterogenous, and socioeconomically deprived communities are disproportionately affected. A broad array of social determinants contributes to the onset and progression of CVD, creating a wide array of interlinked factors. These social determinants of health (SDOH) encompassing 6 key domains: economic stability, education, health care, neighborhood/built environment, community/social context, and food insecurity significantly influence prevalence and outcomes of CVD.^2^ However, despite a heightened focus on longstanding health disparities in the United States, our understanding of the impact of SDOH on CVD remains limited and under-researched.

In an effort to dissect the mechanisms by which SDOH contributes to CVD, public health researchers have introduced a multidisciplinary approach. This involves a blend of epidemiological and translational research to reveal the relationships between SDOH and cardiovascular health. Within this focus of research lies the vital task of quantifying SDOH, a method of characterization that has recently taken center stage. Multiple metrics have been developed to quantify SDOH. This includes collection of objective data across distinct SDOH domains that may impact the health of an individual or community. For example, the Area Deprivation Index, Distressed Communities Index, Community Need Index, and the social vulnerability index (SVI) have all been introduced as potential markers of adversity or social deprivation and vulnerability.^3^, ^4^, ^5^ In contrast to other available indices, the SVI offers granular data on 16 unique social variables at the county and census-level tracts under 4 themes including socioeconomic status, household characteristics, racial and ethnic minority status, and housing type and transportation. Characteristics of these 4 themes are further described in Table 1. Social vulnerability refers to a community's potential susceptibility to adverse effects resulting from natural or human-induced disasters, as well as disease outbreaks. These communities, burdened by their vulnerability, frequently need additional assistance before, during, and after such events. The Agency for Toxic Substances and Disease Registry under the Centers for Disease Control and Prevention leverages the SVI to pinpoint these geographically vulnerable regions.^5^ Accordingly, they can effectively identify the regions that require urgent aid and sustained support in the face of impending or ongoing disasters.

**Table 1:** Characterization of the Social Vulnerability Index

Socioeconomic Status	Household Characteristics	Racial and Ethnic Minority Status (1 of the Following)	Housing Type and Transportation
Unemployed	Single-parent households	Black and African American, not Hispanic or Latino	Mobile homes
Below 150% poverty	Age 17 y or younger	American Indian and Alaska Native, not Hispanic or Latino	Crowding
No health insurance	English language proficiency	Hispanic or Latino (of any race)	Group quarters
No high school diploma	Civilian with a disability	Asian, not Hispanic or Latino	No vehicle
Housing cost burden	Age 65 y or older	Native Hawaiian and other Pacific Islander, not Hispanic or Latino	Multiunit structures
		2 or more races, not Hispanic or Latino	
		Other races, not Hispanic or Latino	

Four underlying themes and 16 components of the social vulnerability index.

While the SVI was initially conceived to assist emergency response planners and public health officials in identifying communities requiring additional support, its application has extended in recent years. Researchers have begun to evaluate its impact across the entire spectrum of CVD care as a marker of global SDOH. The SVI has been found to significantly influence many stages of the CVD trajectory. This ranges from exposure to risk factors and diagnosis, treatment, survivorship care, and ultimately, access to care.^6^, ^7^, ^8^, ^9^, ^10^, ^11^, ^12^, ^13^ Thus, our study aimed to review the existing body of literature that investigates the correlation between the SVI and the continuum of CVD care. By describing the study populations, quality of the studies, and evaluating the use of the SVI, we aimed to identify gaps in the current understanding of the SVI impact on CVD care continuum and highlight potential avenues for future research.

## Methods

Our review was completed in accordance with the PRISMA (Preferred Reporting Items for Systematic Reviews and Meta-Analyses) guidelines.^14^ The primary objective was to identify and map the emerging evidence of SVI's impact on the CVD care continuum, rather than to evaluate the feasibility or effectiveness of specific practices or treatments. Accordingly, we conducted the study using a scoping review approach, which is best suited to assess the breadth of a particular body of literature.^15^

### Eligibility criteria

Studies were deemed eligible for inclusion if they analyzed the impact of the SVI on the CVD care continuum, including observational and qualitative studies. Systematic reviews and meta-analyses may be included for evaluation of their reference lists. We excluded editorials, opinion pieces, and conference abstracts. Populations of interest among the included studies included all age groups and sexes who were diagnosed with CVD or at risk of CVD. We also required that SVI be clearly defined and measured using the Centers for Disease Control and Prevention Agency for Toxic Substances and Disease Registry database. No restrictions were used regarding our assessed outcomes, as these included any aspect of the CVD care continuum, including prevention, diagnosis and prevalence, treatment, and health outcomes (eg, mortality, morbidity, quality of life, patient satisfaction). We included studies written in English. Studies of all quality levels were included, as the purpose of this review is to provide a broad overview of the existing literature, rather than synthesize high-quality evidence.

### Data sources

Seven resources were utilized to search for eligible studies from inception to May 19, 2023. Specifically, we searched Ovid MEDLINE ALL (1946-2023), Embase.com Embase & MEDLINE (1966-2023), Scopus, EBSCOhost CINAHL Plus with Full Text, and ProQuest Environmental Science Collection (1960-2023). In our preliminary exploration of the topic, we identified publications that would not get captured with the search because the search terms/concepts were in the full text. We therefore decided to also utilize 2 resources that search within the full text: PubMed Central and Google Scholar. References of included studies were screened for studies of relevance. Search results were managed by using the EndNote 20 software program where duplicates were identified and removed from the pool of search results available for screening. The search strategies for this review are provided in the Supplemental Appendix.

### Study selection

Two independent investigators initiated the screening process. This stage involved a review of the titles and abstracts of the search results, aiming to identify research exploring the influence of SVI on various aspects of the CVD care continuum. All studies deemed potentially relevant based on their title and abstract underwent full-text retrieval. The same 2 investigators independently assessed each full-text article, determining its suitability for inclusion in the scoping review. The evaluation was based on a predetermined inclusion and exclusion criteria. In instances of disagreement between the 2 primary investigators, or when uncertainties arose, a third independent investigator was consulted.

### Data extraction

The extraction of data from each study included in the review was conducted by the same 2 investigators that conducted the study selection. Each investigator thoroughly reviewed and extracted pertinent information from each article. Upon the completion of individual data extraction, the 2 investigators compiled their respective findings. They then cross-checked each other's results to ensure accuracy and consistency in the information obtained. The data extraction focused on several key variables deemed significant to our research question. These included the study design, data sources utilized, specific use and operationalization of the SVI, patient population studied, research question or aim of the study, other SDOH measured, quality of the study, and the outcomes measured in the study.

### Quality assessment

The assessment of the quality of the included studies was conducted using the STROBE (Strengthening the Reporting of Observational Studies in Epidemiology) checklist.^16^ An internationally recognized tool, the STROBE checklist was developed by a global collaborative initiative to support researchers in enhancing the transparency and comprehensiveness of their observational studies, including case-control, cross-sectional, and cohort designs. The rationale behind selecting this checklist was its ability to offer robust guidelines that provide sufficient clarity. This clarity, in turn, allows effective appraisal and interpretation of findings, ensuring the integrity and reliability of the analysis.

## Results

**Figure 1:**
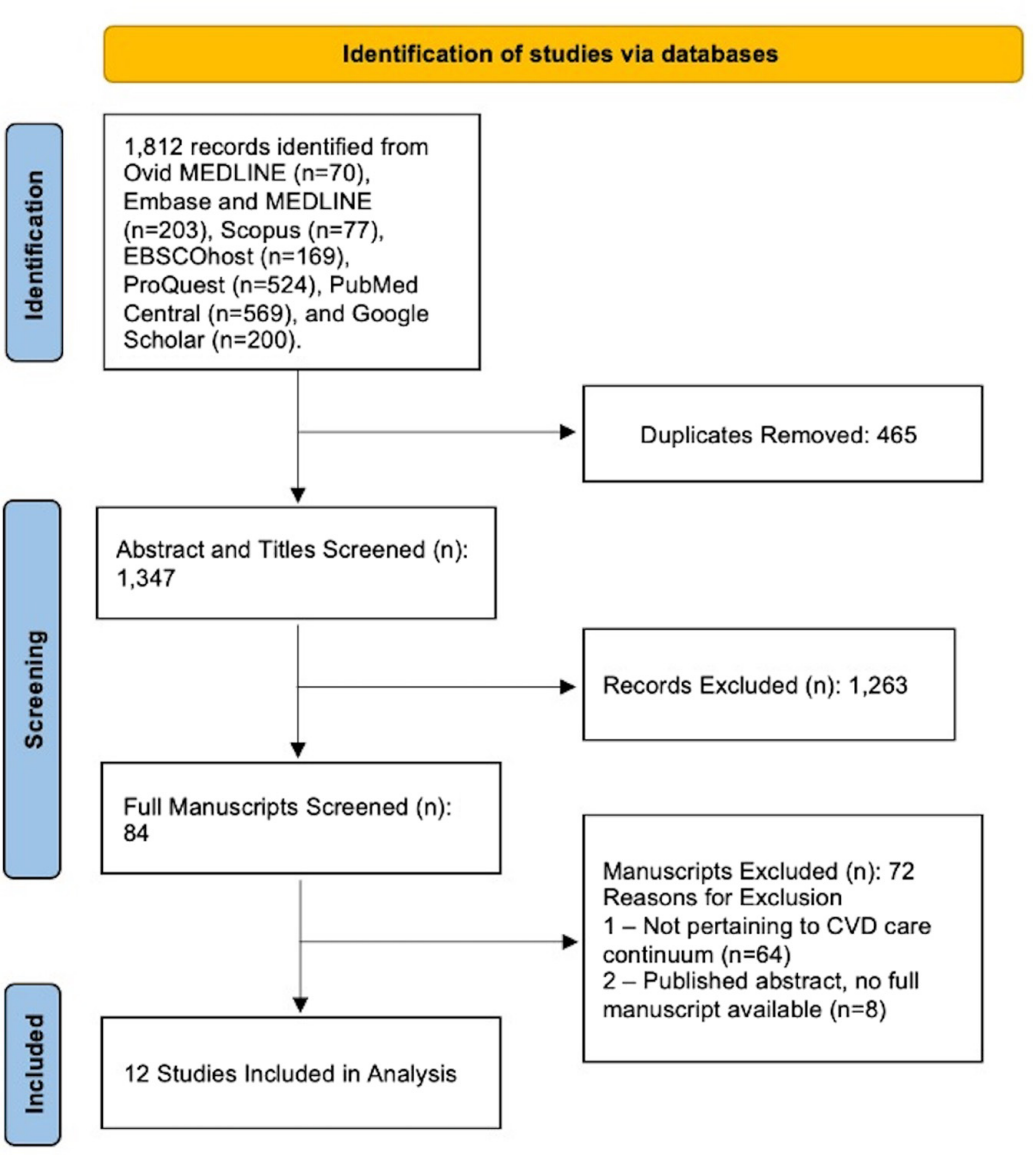
PRISMA Flow Diagram Systematic approach to study retrieval for the scoping review. CVD = cardiovascular disease; PRISMA = Preferred Reporting Items for Systematic Reviews and Meta-Analyses.

Figure 1 depicts the flow of records from the search yield through the study selection process. The initial search yielded a total of 1,812 records from which 465 duplicates were identified leaving the number of records available for study selection to be 1,347. After completing the title/abstract screening of records for relevance, 1,263 records were excluded, and 84 records moved forward to the full-text screening phase. In the full-text screening phase, studies were evaluated based on the inclusion/exclusion criteria. The majority of excluded studies were due to assessment of various SDOH measures that did not include the SVI, or the studies evaluated the impact of the SVI on outcomes unrelated to the CVD care continuum. A total of 12 studies were ultimately selected for inclusion in our scoping review. Detailed summaries of these studies can be found in Table 2.

**Table 2:** 12 Included Studies

Study	SVI Data Release, Year	Study Design	Research Question	Outcomes	STROBE Score (0-22)
Neighborhood-level Social Vulnerability and Prevalence of Cardiovascular Risk Factors and Coronary Heart Disease	2018 SVI release	Cross-sectional design	What is the association between SVI and prevalence of CVD risk factors and coronary heart disease in the US?	Geographic disparities among risk factor prevalence and coronary heart disease diagnosis prevalence	22
Impact of Social Vulnerability on Comorbid Cancer and Cardiovascular Disease Mortality in the United States	2018 SVI release	Cross-sectional design	What is the impact of the SVI on CVD mortality, cancer mortality, and both comorbid conditions?	Crude- and age-adjusted mortality rates (and risk ratios) in areas with varying SVI rankings	22
Social Vulnerability and Excess Mortality in the COVID-19 Era	2018 SVI release	Cross-sectional design	What are the trends in excess all-cause and cardiovascular mortality in the COVID-19 era, sorted by indexes of SVI rankings?	Excess CVD and all-cause death counts and mean relative differences in relation the varying SVI rankings	18
Social Vulnerability and Premature Cardiovascular Mortality Among US Counties, 2014 to 2018	2018 SVI release	Cross-sectional design	Is there an association between SVI and premature CVD mortality?	Age-adjusted mortality among SVI quartiles and corresponding risk ratios	22
Association Between Social Vulnerability Index and Cardiovascular Disease: A Behavioral Risk Factor Surveillance System Study	2018 SVI release	Retrospective-cohort analysis	Is there an association between SVI and prevalence of atherosclerotic cardiovascular disease and cardiovascular comorbidities?	Prevalence of cardiovascular comorbidities and atherosclerotic cardiovascular disease based on SVI rankings	22
Social Vulnerability and Location of Death in Heart Failure in the United States	Not recorded	Cross-sectional analysis	Are populations with greater social vulnerability less likely to utilize hospice care and more likely to die in the hospital?	Geographic mortality rates at different locations in the U.S. based on SVI rankings	20
County-Level Social Vulnerability is Associated With In-Hospital Death and Major Adverse Cardiovascular Events in Patients Hospitalized With COVID-19: An Analysis of the American Heart Association COVID-19 Cardiovascular Disease Registry	2018 SVI release	Cross-sectional design	Does the SVI have an impact on in-hospital outcomes in those with COVID-19?	County-level rates of in-hospital all-cause mortality and major adverse cardiovascular events in relation to COVID-19	22
The Association Between Neighborhood Social Vulnerability and Cardiovascular Health Risk Among Black/African American Women in the InterGEN Study	2018 SVI release	Cross-sectional design	Is there an association between the SVI and cardiovascular risk factors among Black women?	U.S. neighborhood-level prevalence of hypertension, obesity, and depression	20
State-Level Social Vulnerability Index and Healthcare Access in Patients With Atherosclerotic Cardiovascular Disease (from the BRFSS Survey)	2018 SVI release	Cross-sectional design	Is there an association between the SVI and access to health care among individuals with a history of atherosclerotic CVD?	State-level data, based on SVI rankings, to having a PCP, health care coverage, duration since last routine check-up, inability to see a doctor because of care, cost-related medication nonadherence, delay In accessing health care	18
Associations of Social Vulnerability Index With Pathologic Myocardial Findings at Autopsy	2018 SVI release	Cross-sectional design	Is there an association between the SVI and physiological stressors on myocardial-level pathology?	Myocardial tissue-level outcomes, based on SVI rankings, including any coronary atherosclerosis, myocardial fibrosis, and/or myopericardial inflammation	22
Association of participation in Cardiac Rehabilitation with Social Vulnerability Index: The behavioral risk factor surveillance system	Not recorded	Cross-sectional design	Is there an association between the SVI and use of cardiac rehabilitation following acute myocardial infarction?	Use of cardiac rehabilitation services by varying SVI rankings	22
Social Vulnerability Indices as a Risk Factor for Heart Failure Readmissions	Not recorded	Retrospective cohort study	Is there an association between the SVI and hospital readmission rates in patients with heart failure?	Rates of heart failure readmissions by varying SVI rankings	19

Summary and characteristics of each included study in the scoping review.

CVD = cardiovascular disease; PCP = primary care physician; STROBE = Strengthening the Reporting of Observational Studies in Epidemiology; SVI = social vulnerability index.

### Study populations and outcome measures

**Figure 2:**
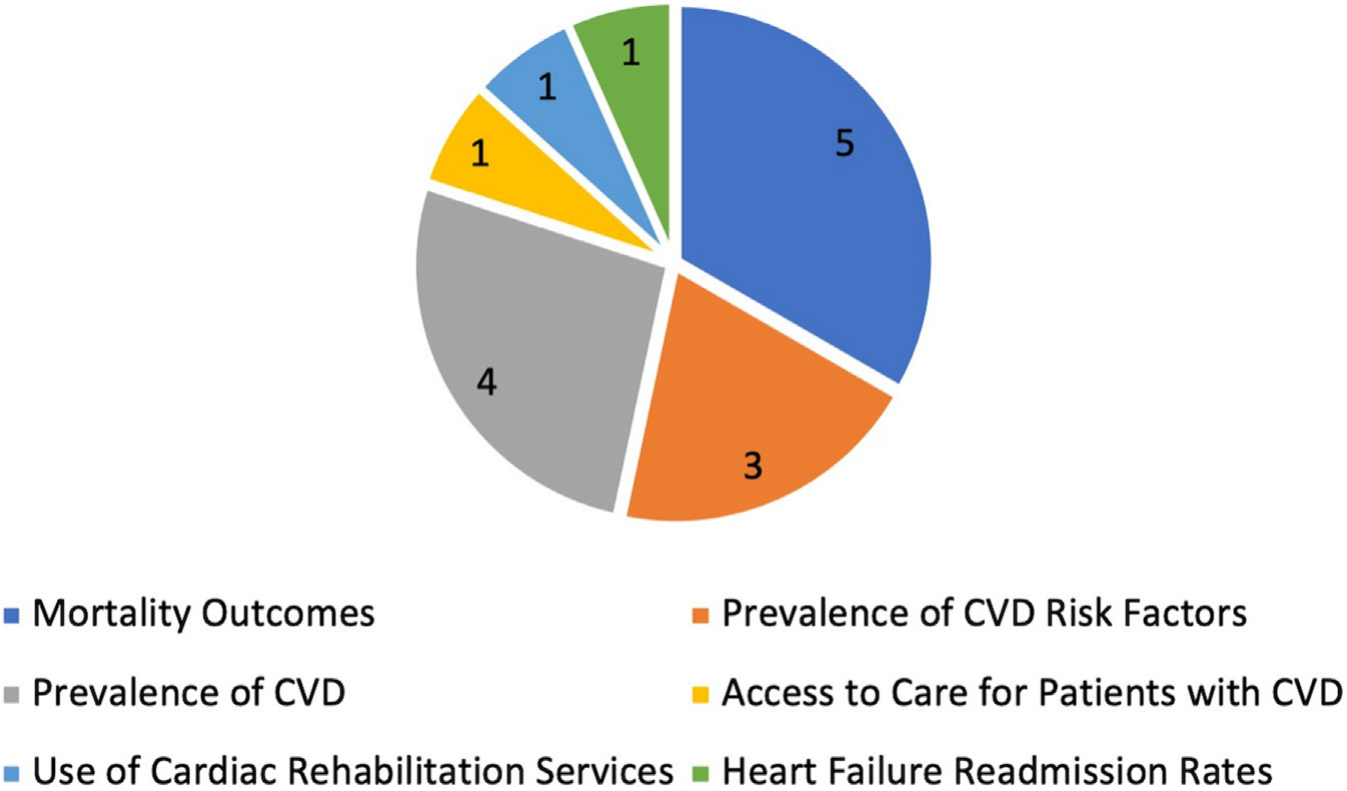
Evaluation of SVI in the CVD Care Continuum Number of studies that evaluated the corresponding component of the CVD care continuum. CVD = cardiovascular disease; SVI = social vulnerability index.

Our analysis encompassed a diverse range of study populations and outcome measures, providing a comprehensive view of the existing research landscape on the SVI in relation to CVD. In 5 of the selected studies, the primary objective was to evaluate the influence of the SVI on mortality outcomes related to CVD.^8^^,^^9^^,^^17^, ^18^, ^19^ Three studies probed the impact of the SVI on CVD risk factors.^6^^,^^7^^,^^10^ Four studies examined the association between the SVI and the prevalence of CVD.^7^^,^^9^^,^^10^^,^^12^ One study focused on the association between the SVI and access to health care in patients diagnosed with atherosclerotic CVD.^20^ One study explored the association between the SVI and the utilization of cardiac rehabilitation services.^21^ One study investigated the impact of the SVI on hospital readmission rates among patients who were recently discharged following an episode of acutely decompensated heart failure.^11^ Overall, these studies provide a multifaceted perspective on how the SVI can influence different aspects of the CVD care continuum (Figure 2).

### Utilized data sources

Within the included studies, there was a broad spectrum of data sources used, ranging from publicly accessible data repositories to institutional records and survey data. Two studies relied on the Behavioral Risk Factor Surveillance System database to procure data on CVD risk factor prevalence and coronary heart disease prevalence.^7^^,^^10^ Another study made use of data from the 2010 American Community Survey to establish urban-rural classifications, allowing for a stratified analysis of their results.^7^ Four studies utilized the multiple causes of death database to obtain mortality data.^8^^,^^17^, ^18^, ^19^ Three studies utilized the Behavioral Risk Factor Surveillance System database to obtain a wealth of demographic information and other SDOH.^10^^,^^20^^,^^21^ These data included patient age, race/ethnicity, employment status, educational status, tobacco use, income level, and indicators of health care access. Institutional data records provided a direct and detailed source of patient information in 2 studies.^11^^,^^12^ One study leveraged the American Heart Association COVID-19 CVD Registry to inform their analysis.^9^ Lastly, another study conducted a secondary analysis of the InterGEN Study to secure patient information relevant to their objectives.^6^

### Use of the SVI

SVI data are available at both the census-tract and county level, as each residential address in the United States is linked with a unique 15-digit geographic identifier which includes a state code, county code, census tract code, and, where relevant, a census block code. This enables patient data to be tied with specific SVI data, providing a clear geographical context for the analysis. Census tracts, as aggregates of county subdivisions, were utilized as the unit of SVI data in 4 of the studies.^6^^,^^7^^,^^12^^,^^21^ One of these studies further leveraged the census-tract level data to ascertain neighborhood vulnerability.^6^ Conversely, 6 studies drew on SVI data at the county level.^8^, ^9^, ^10^^,^^17^, ^18^, ^19^ Three studies employed state-level SVI rankings,^10^^,^^20^^,^^21^ while 1 study utilized SVI data at the zip code level.^11^

The SVI offers a comprehensive ranking for each geographical region, comprising 16 social factors grouped under 4 themes: household characteristics, socioeconomic status, racial and ethnic minority status, and housing type and transportation. These themes, individually and collectively, are assigned percentile rankings from 0 to 1 to indicate the level of social vulnerability, with 0 representing the least vulnerable and 1 the most vulnerable. Studies can assess the impact of SVI on CVD using either the aggregate SVI ranking, encompassing all 4 themes, or by focusing on individual themes. All 12 studies employed the overall SVI aggregate percentile ranking to assess its impact on CVD.^6^, ^7^, ^8^, ^9^, ^10^, ^11^, ^12^^,^^17^, ^18^, ^19^, ^20^, ^21^ Five of these studies went further by analyzing the individual SVI themes to determine their specific impacts on CVD.^6^^,^^7^^,^^9^^,^^10^^,^^18^

Regarding the interpretation of SVI rankings, 6 studies divided the overall percentile rankings into quartiles,^7^, ^8^, ^9^^,^^17^, ^18^, ^19^ and 3 studies similarly segmented the SVI into tertiles.^10^^,^^20^^,^^21^ Seven studies treated the SVI as a continuous variable,^6^, ^7^, ^8^^,^^11^^,^^12^^,^^19^^,^^21^ while 1 study used the SVI as a categorical variable without subdividing its overall percentile rankings into quartiles or tertiles.^6^

### Associations between SVI and CVD

In all 12 studies, greater SVI was statistically associated with multiple components of the CVD continuum. This included increased premature cardiovascular death,^18^ higher likelihood of dying from heart failure at home or an inpatient facility compared to nursing homes,^19^ decreased access to cardiac rehabilitation and health care access,^20^^,^^21^ increased CVD risk and coronary heart disease prevalence,^6^^,^^7^^,^^10^ higher odds of myocardial fibrosis on autopsy,^12^ higher cardio-oncology-related death,^8^ and poor outcomes including readmissions and mortality.^9^^,^^11^^,^^17^

### Study quality

All included studies were appraised through utilization of the STROBE checklist. The score ranged from 0 to 22 based on the number of included items. The majority of our studies completed all items on this checklist with a score of 22.^7^, ^8^, ^9^, ^10^^,^^12^^,^^18^^,^^21^ Two studies scored 20^6^^,^^19^, 1 study scored 19,^11^ and 2 studies scored 18.^17^^,^^20^

## Discussion

In this scoping review, we comprehensively explored the current body of literature examining the relationship between the SVI and the CVD care continuum (Central Illustration). We demonstrate the versatile utility of the SVI in examining various facets of CVD, from risk factors and prevalence to health care access and outcomes, including mortality. The ways in which the SVI was employed also varied, with all studies using the aggregate index and a few of which investigated the individual thematic components. This provided a multidimensional perspective on the role social vulnerability plays in CVD care.

**Central Illustration:**
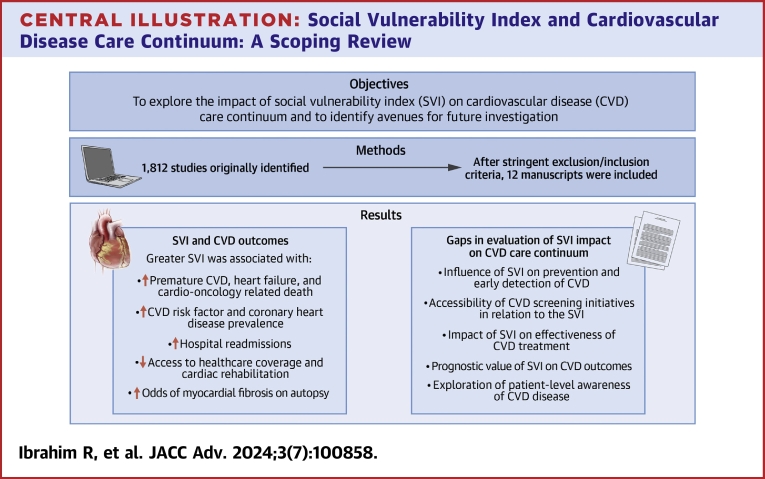
Social Vulnerability Index and Cardiovascular Disease Care Continuum: A Scoping Review

Social vulnerability focuses on a comprehensive approach to measure social circumstances in communities in the United States that differs in many ways from SDOH (conditions related to where people are born, live, age, and work) and socioeconomic status (social status, income, education).^22^, ^23^, ^24^, ^25^ The SVI has been associated with many poor health outcomes which include mortality, disability, and cognitive impairment.^13^^,^^24^, ^25^, ^26^ Chronic conditions were also found to be more frequent in populations with a higher SVI, which include depression and anxiety, CVD, and obesity.^27^ Given that aspects of life control and social engagement are quantified in the SVI, these populations are expected to have higher rates of smoking, physical inactivity, heavy drinking, and limited access to health care and healthy foods.^28^, ^29^, ^30^ These unique characteristics of the SVI support its use when evaluating social aspects of cardiovascular care in the United States.

**Figure 3:**
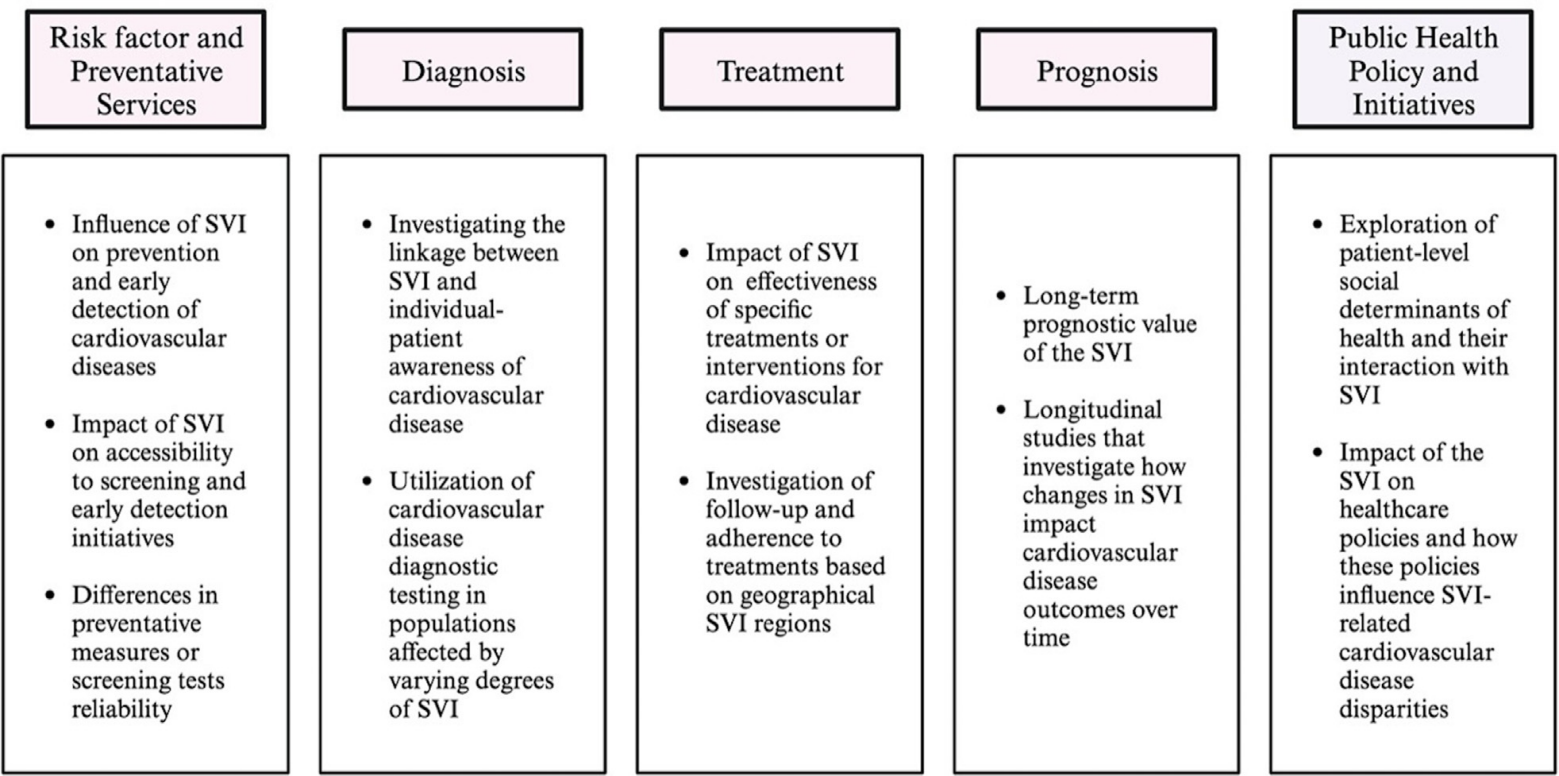
Gaps in Understanding Regarding the Impact of SVI on CVD Care Continuum Areas of potential investigation regarding the impact of the SVI on the CVD care continuum. CVD = cardiovascular disease; SVI = social vulnerability index.

One of the key goals of our study was to identify the knowledge gaps regarding the impact of social vulnerability on the CVD care continuum (Figure 3). Among the 12 identified studies, there was a lack of emphasis on the influence of SVI on prevention and early detection of CVD, including accessibility and effectiveness of preventive screenings and early detection initiatives. There was also a lack of understanding regarding the effectiveness of specific treatments or interventions for CVD. Complementing SVI, an exploration of patient-level SDOH and their interaction with SVI might enrich our understanding of individual health outcomes. This also includes investigating the linkage between SVI and individual-patient awareness of CVD. Furthermore, most included studies are based on cross-sectional analyses, suggesting the need for longitudinal studies that investigate how changes in SVI, or counties among varying SVI rankings, impact CVD outcomes over time. Lastly, given the profound impact of the SVI on CVD care continuum, further investigation into the impact of the SVI on health care policies and how these policies influence SVI-related CVD disparities would be warranted. These findings aim to empower population-health researchers to identify these missing components of SVI in CVD care continuum. Additionally, with rapid advancements of machine learning and artificial intelligence technologies, there exists unprecedented opportunities to integrate the SVI into large population-health and electronic medical record databases. With the use of geocoding applications, this would allow epidemiologists and researchers to analyze the spatial distribution of the SVI components alongside clinical health records, paving the way for development of polysocial risk scoring systems. These scores would be instrumental in targeting the multifaceted nature of health risks faced by individuals.

The impact of SDOH on cardiovascular outcomes is poorly understood as a result of the inadequate attention to socially vulnerable groups. Furthermore, this is complicated by contemporary reimbursement models that mitigate the need to document and use SDOH in clinical decision-making, time and resources promoting collection of SDOH data in real-world databases, and lack of application of quantitative analytic methods to assess the impact of SDOH on a population scale.^31^ SDOH definitions consider a range of conditions in the environments where people are born, live, and socialize, acknowledging their potential effects on health and life quality. However, these definitions may not adequately capture critical social factors like discrimination, stigma, and marginalization. Elements such as ethnic residential segregation, the quality of neighborhood infrastructure, accessibility to small grocery outlets, personal perceptions of safety, job-related stress, and feelings of loneliness or social isolation have all been tied to overall cardiovascular well-being.^32^ These findings deepen our understanding of the social phenomena that recurrently impact the care continuum of CVD. Enhancing our understanding of the social phenomena directly affecting these groups could guide the development of targeted strategies for social improvement and policy reform, ultimately contributing to enhanced cardiovascular health outcomes.

While broader social reforms are essential for addressing the long-term impacts of social vulnerability on cardiovascular health, several initiatives can improve the quality of care among these vulnerable populations. Clinicians may undergo further training to comprehend and recognize the effects of social vulnerability, fostering an informed approach to care. Furthermore, tailoring individualized care plans that factor in these vulnerabilities may lead to more effective interventions and adherence. Enhancing collaborations between hospital systems and community outreach organizations through awareness and health screening events may improve medical literacy. Finally, directing resources such as social workers, community health works, and other health care professionals to high vulnerability regions may narrow the existing care gap.

### Study Limitations

Our study is not without limitations. Given the population-level design of the included studies, these findings are unlikely to be exclusively applicable to individual-level data. Our preliminary exploration of the topic revealed potentially relevant literature that would not get captured with searching of records in databases but had the pertinent information in the full text. While we made an effort to search resources that provide full-text searching, it is possible that studies were missed relating to this factor. Additionally, studies that evaluated the impact of SVI on populations who were not diagnosed or at risk of CVD were excluded; however, studies may have been inadvertently excluded from this analysis if they were not explicitly identified during the screening process. Lastly, studies with statistically significant findings are more likely to be published; hence, publication bias remains a possibility.

## Conclusions

Our scoping review explored the relationship between the SVI and the continuum of CVD care. The SVI has been evaluated for its impact on mortality outcomes, CVD risk factors, disease prevalence, access to health care, utilization of cardiac rehabilitation services, and hospital readmission rates, demonstrating the versatile use of the SVI as a comprehensive metric of social vulnerability. Gaps in understanding the impact of SVI were identified, primarily among CVD prevention, early detection, and effectiveness of treatment options.

PERSPECTIVES **COMPETENCY IN PATIENT CARE:** The social vulnerability index has been shown to influence many different stages of the cardiovascular disease trajectory, emphasizing the importance of considering social determinants when managing these patients.

**TRANSLATIONAL OUTLOOK 1:** A diverse array of populations, outcome measures, data sources, and applications of the social vulnerability index were seen, emphasizing the versatile utility of the social vulnerability index in examining the multifaceted nature of the cardiovascular disease care continuum.

**TRANSLATIONAL OUTLOOK 2:** Gaps in knowledge regarding the impact of the social vulnerability index on cardiovascular disease care continuum exist, primarily related to prevention, early detection, and effectiveness of treatment modalities, highlighting potential avenues for research.

## Funding support and author disclosures

This research did not receive any specific grant from funding agencies in the public, commercial, or not-for-profit sectors. The authors have reported that they have no relationships relevant to the contents of this paper to disclose.
