# Supplementary material for: Social Vulnerability Index and Cardiovascular Disease Care Continuum: A Scoping Review
Source: JACC Adv. 2024 Mar 6;3(7):100858. doi: 10.1016/j.jacadv.2024.100858 (PMC11312302; doi:10.1016/j.jacadv.2024.100858)
Supplement: Supplemental data [file mmc1.docx]

**SUPPLEMENTAL APPENDIX**

**Search Strategies**

**Ovid MEDLINE.** Search strategy used on Ovid MEDLINE.

| **#** | **Searches** |
| --- | --- |
| 1 | social vulnerability/ |
| 2 | (("social vulnerability" adj3 (index or indices)) or "cdc svi").af. |
| 3 | ((neighborhood* or "zip code*" or county or counties) adj3 vulnerability).af. |
| 4 | or/1-3 |
| 5 | exp cardiovascular system/ or exp cardiovascular diseases/ or exp cardiovascular abnormalities/ or exp cardiovascular surgical procedures/ or cardiac rehabilitation/ or exp cardiology/ or exp heart disease risk factors/ |
| 6 | (atherosclerosis or atherosclerotic or atrial or cardiac or cardiovascular or cardiology or cardiomyopathy or cardiometabolic or coronary or heart or hypertension or "blood pressure" or myocardial or pericardial or ventricular).af. |
| 7 | 5 or 6 |
| 8 | 4 and 7 |

**Embase.com Embase & MEDLINE.** Search strategy used for Embase and MEDLINE.

| No. | Query |
| --- | --- |
| #8 | #4 AND #7 |
| #7 | #5 OR #6 |
| #6 | atherosclerosis OR atherosclerotic OR atrial OR cardiac OR cardiovascular OR cardiology OR cardiomyopathy OR cardiometabolic OR coronary OR heart OR hypertension OR 'blood pressure' OR myocardial OR pericardial OR ventricular |
| #5 | 'cardiovascular system'/exp OR 'cardiovascular disease'/exp OR 'cardiovascular malformation'/exp OR 'cardiovascular procedure'/exp OR 'cardiovascular mortality'/de OR 'heart rehabilitation'/de OR 'cardiology'/exp OR 'cardiovascular risk factor'/exp OR 'cardiometabolic risk factor'/de OR 'cardiovascular risk'/exp OR 'cardiometabolic risk'/de |
| #4 | #1 OR #2 OR #3 |
| #3 | (neighborhood* OR 'zip code*' OR county OR counties) NEAR/3 vulnerability |
| #2 | ('social vulnerability' NEAR/3 (index OR indices)) OR 'cdc svi' |
| #1 | 'social vulnerability index'/de OR 'social vulnerability'/de |

**Scopus:**

TITLE-ABS-KEY (("social vulnerability" W/3 (index or indices)) OR "cdc svi" OR ((neighborhood* OR "zip code*" OR county OR counties) W/3 vulnerability)) AND
TITLE-ABS-KEY (atherosclerosis OR atherosclerotic OR atrial OR cardiac OR cardiovascular OR cardiology OR cardiomyopathy OR cardiometabolic OR coronary OR heart OR hypertension OR "blood pressure" OR myocardial OR pericardial OR ventricular)

**EBSCOhost CINAHL Plus with Full Text.** Search strategy for EBSCOhost CINAHL Plus.

| **#** | **Query** |
| --- | --- |
| S8 | S4 AND S7 |
| S7 | S5 OR S6 |
| S6 | (atherosclerosis or atherosclerotic or atrial or cardiac or cardiovascular or cardiology or cardiomyopathy or cardiometabolic or coronary or heart or hypertension or "blood pressure" or myocardial or pericardial or ventricular) |
| S5 | (MH "Cardiovascular Risk Factors+") OR (MH "Cardiovascular Diseases+") OR (MH "Cardiovascular Abnormalities+") OR (MH "Surgery, Cardiovascular+") OR (MH "Cardiovascular System+") OR (MH "Diagnosis, Cardiovascular") OR (MH "Cardiac Patients") OR (MH "Rehabilitation, Cardiac+") |
| S4 | S1 OR S2 OR S3 |
| S3 | ((neighborhood* or "zip code*" or county or counties) N3 vulnerability) |
| S2 | ("social vulnerability" N3 (index or indices)) OR "cdc svi" |
| S1 | ((MH "Social Determinants of Health") AND (MH "Residence Characteristics+")) OR ((MH "Social Determinants of Health") AND (MH "Vulnerability")) OR ((MH "Vulnerability") AND (MH "Residence Characteristics+")) |

**ProQuest Environmental Science Collection (Environmental Science Index and Environmental Science Database):**

(("social vulnerability" NEAR/3 (index OR indices)) OR "cdc-svi" OR ((neighborhood* OR "zip code*" OR county OR counties) NEAR/3 vulnerability)) AND (subject(atherosclerosis) OR subject(atrial) OR subject(cardiac) OR subject(cardiovascular) OR subject(cardiology) OR subject(cardiomyopathy) OR subject(coronary) OR subject(heart) OR subject(hypertension) OR subject(blood pressure) OR subject(myocardial) OR subject(pericardial) OR subject(ventricular) OR (atherosclerosis OR atherosclerotic OR atrial OR cardiac OR cardiovascular OR cardiology OR cardiomyopathy OR cardiometabolic OR coronary OR heart OR hypertension OR "blood pressure" OR myocardial OR pericardial OR ventricular))

**PubMed Central:**

(social vulnerability [MeSH Terms] OR "social vulnerability index" [all]) AND (cardiovascular system [MeSH Terms] OR cardiovascular diseases [MeSH Terms] OR cardiovascular abnormalities [MeSH Terms] OR cardiovascular surgical procedures [MeSH Terms] OR cardiac rehabilitation [MeSH Terms] OR cardiology [MeSH Terms] OR heart disease risk factors [MeSH Terms] OR (atherosclerosis [all] OR atherosclerotic [all] OR atrial [all] OR cardiac [all] OR cardiovascular [all] OR cardiology [all] OR cardiomyopathy [all] OR cardiometabolic [all] OR coronary [all] OR heart [all] OR hypertension [all] OR "blood pressure" [all] OR myocardial [all] OR pericardial [all] OR ventricular [all]))

**Google Scholar:**

Searched by keywords in Harzing's Publish or Perish 8 and retrieved the first 200 search results

"social vulnerability index"+atherosclerosis|atherosclerotic|atrial|cardiac|cardiovascular|cardiology|cardiomyopathy|cardiometabolic|coronary|heart|hypertension|"blood pressure"|myocardial|pericardial|ventricular
